# Does testosterone mediate the relationship between vitamin D and prostate cancer? A systematic review and meta-analysis protocol

**DOI:** 10.1186/s13643-018-0908-1

**Published:** 2019-02-12

**Authors:** Luke A. Robles, Karen Dawe, Richard M. Martin, Julian P. T. Higgins, Sarah J. Lewis

**Affiliations:** 1 Bristol Medicine School, Population Health Sciences, University of Bristol, Bristol, England; 2 MRC Integrative Epidemiology Unit (IEU), University of Bristol, Office OG25, Oakfield House, Oakfield Grove, Bristol, BS8 2BN England; 3 National Institute for Health Research (NIHR) Bristol Biomedical Research Centre, University of Bristol, Bristol, England

**Keywords:** Vitamin D, Testosterone, Prostate cancer, Mechanistic pathway, Systematic review

## Abstract

**Background:**

Evidence from studies on prostate cancer progression have identified vitamin D to be a potentially important nutrient. However, the World Cancer Research Fund and American Institute for Cancer Research have reported the quality of this evidence to be limited and warrant further investigation. We plan to use the recently developed WCRF International/University of Bristol mechanistic systematic review framework to determine whether the observed association between vitamin D and prostate cancer exists through a plausible biological pathway.

**Methods:**

This protocol sets out how we will perform a systematic review of the literature in human and animal studies. We will search the electronic databases MEDLINE, EMBASE, PubMed, and BIOSIS Citation Index without restrictions on year of publication or language. We will extract data from observational and experimental studies examining two inter-linked pathways in the relationship between vitamin D and prostate cancer progression: (1) vitamin D and testosterone, and (2) testosterone and prostate cancer progression. We focus on testosterone as its actions form a potentially novel intermediate mechanism that was identified via our online literature mining tools. The outcomes of interest include incidence or prevalence of prostate cancer, measures of prostate cancer progression (including biochemical recurrence, local, or distal metastases), and prostate cancer-specific mortality. We will assess study quality and the level of certainty of the evidence. We will analyse data where possible, using meta-analysis with forest plots or albatross plots; otherwise, a narrative synthesis will be performed.

**Discussion:**

To our knowledge, this will be the first systematic synthesis of the evidence underpinning the vitamin D-testosterone-prostate cancer mechanistic pathway. The results of the review may inform future research, intervention trials, and public health messages.

**Electronic supplementary material:**

The online version of this article (10.1186/s13643-018-0908-1) contains supplementary material, which is available to authorized users.

## Background

Prostate cancer is the second most commonly diagnosed cancer and the fifth leading cause of cancer death in men worldwide [1]. The causes of prostate cancer are not well understood, but well-established risk factors are older age [2], Afro-Caribbean or West African origin [3], and having a first-degree relative (father, brother) diagnosed with the disease [4]. None of these factors are modifiable or controllable through intervention. Other putative risk factors for prostate cancer include obesity, physical activity, and diet. These lifestyle factors are amenable with intervention but definitive evidence that they are causally associated with prostate cancer is lacking.

The World Cancer Research Fund Continuous Update Project [5] reviewed the literature on certain nutrients associated with prostate cancer including vitamin D, and found the evidence was limited and inconclusive regarding their effects on prostate cancer. However, the vitamin D receptor is active in many tissues, including prostate tissue [6], and ecologic studies suggest that in regions where sun exposure (the major source of vitamin D) is high there is a reduced risk of prostate cancer [7, 8]. Individual-level observational studies report inconsistent results, with both high and low vitamin D concentrations associated with an increased risk of prostate cancer [9, 10]. These inconsistencies may be explained by small sample sizes or confounders of the vitamin D-prostate cancer relationship. With regards to prostate cancer progression (rather than incidence), there is randomised controlled trial evidence suggesting a modest effect of interventions to increase vitamin D using supplements on measures of disease progression (PSA levels, decrease in number of positive cores, and Gleason score), among men with low and intermediate stage prostate cancer [11, 12].

Vitamin D is a precursor to the steroid hormone calcitriol, which regulates calcium and phosphate and has an important role in bone mineralisation [13]. The majority of vitamin D is obtained from exposure to sunlight which is synthesised through the skin in the form of cholecalciferol (vitamin D_3_). A smaller proportion of vitamin D comes from diet as ergocalciferol (vitamin D_2_). Vitamin D is converted into calcitriol through two hydroxylation steps: first, cholecalciferol is hydroxylated in the liver into 25-hydroxy vitamin D; second, circulating 25-hydroxy vitamin D is hydroxylated in the kidney to produce calcitriol (1,25(OH)_2_D) [13].

Vitamin D increases differentiation and apoptosis, and decreases proliferation and metastasis in prostate cancer cells [14]. However, identifying which mechanism explains the association between vitamin D and prostate cancer, and systematically reviewing the evidence for this mechanism is complex. We, therefore, plan to use a recently developed two-stage methodology (the WCRF International/University of Bristol methodology) to synthesise the evidence from mechanistic studies [15]. We have already carried out step 1 of this process, in which we have used two web-based text mining tools to generate our mechanistic hypothesis. TeMMPo (Text Mining for Mechanism Prioritisation) [16] uses MeSH descriptors to quantify the literature on predefined intermediate concept terms between an exposure and an outcome through co-occurrence (i.e. two concepts that occur frequently together in the same article). TeMMPo provides a priority score to assist with identifying novel intermediate concept terms that have a substantial literature resource. MELODI (Mining Enriched Literature Objects to Derive Intermediates) [17] uses MeSH and free text terms to identify overlapping concept terms between two custom article sets. We first used MELODI to yield novel intermediate concept terms, which were then entered into TeMMPo to assist with prioritising terms.

Two co-authors reviewed the first 20 highest ranked intermediate terms produced by TeMMPo and agreed on a potential mechanism using a process of elimination. Seven of the highest ranked terms were eliminated; four terms were too broad (i.e. ribonucleic acid (RNA), RNA messenger, CD4 (gene), nicotinamide adenine dinucleotide), and three terms related to endogenous retroviruses, which have no well-known drug targets nor are amenable with behavioural intervention. The highest ranked term was a gene (i.e. HSD3B1) involved in androgen metabolism followed by testosterone (ranked ninth) after eliminating the other seven terms. Testosterone was, therefore, chosen as the mechanism for investigation.

Testosterone is a male sex hormone that is produced by the testis and adrenal glands, with a critical role in driving cell division in the prostate. Testosterone binds to proteins (sex hormone-binding globulin and albumin) in the blood whilst a small proportion of it is unbound (free testosterone); total testosterone concentrations refer to the combination of bound and free testosterone. Normal total testosterone levels in healthy men are between 300 and 1050 ng/dl. The androgen hypothesis [18] suggests prostate cancer onset or progression is driven by androgens, which is supported by animal models [19] and the observation that castration or high-dose oestrogen therapy to reduce serum testosterone subsequently reduces metastatic prostate cancer. Therapeutic or surgical (castration) androgen deprivation is used clinically to reduce testosterone production in the treatment of prostate cancer.

The relationship between vitamin D and testosterone is not well-understood, although there is randomised placebo controlled trial [20] and observational [21, 22] evidence of a positive relationship between vitamin D and both total and free testosterone, suggesting testosterone is a plausible biological intermediate on the mechanistic pathway between vitamin D and prostate cancer progression. A review of mechanistic studies is, therefore, warranted to support the existence of this relationship.

Our systematic review aims to synthesise the evidence from human and animal studies to investigate whether testosterone is a causal intermediate in the mechanistic pathway between vitamin D and prostate cancer.

As the potential importance of this relationship is only just emerging, few studies are likely to assess both vitamin D and testosterone in relation to prostate cancer. Therefore, relevant indirect evidence will be drawn from studies of the two pathways: (1) studies linking vitamin D to testosterone; and (2) studies linking testosterone to prostate cancer.

## Methods

### Standards of reporting

This protocol was written in accordance with the Preferred Reporting Items for Systematic review and Meta-Analysis Protocols (PRISMA-P) 2015 statement [23]. A populated checklist for this review protocol has been provided in Additional file 1.

### Inclusion and exclusion criteria

Studies must meet the following criteria for the two pathways addressed in the review.

#### Vitamin D-testosterone criteria

- Participants: human or animal—with measures of vitamin D as the exposure and testosterone (free or total testosterone) as the outcome;
- Exposures: any duration, frequency, and dose of vitamin D, including nutrition supplements, and sunlight exposure;
- Outcome: serum or plasma levels of total and/or free testosterone.

#### Testosterone-prostate cancer criteria

- Participants: human or animal—with measures of testosterone (free or total testosterone) as the exposure and prostate cancer as the outcome;
- Exposure: any duration, frequency, and dose of testosterone, including therapeutic use for non-cancerous conditions;
- Outcomes: incidence or prevalence of prostate cancer, number and size of tumour, measures of prostate cancer progression (including biochemical recurrence, local or distal metastases) and prostate cancer-specific mortality;
- Observational studies (cohort, case-control) and experimental studies (randomised controlled trials, cross-over studies) will be eligible for the review.

The following studies will be excluded:

- Studies investigating treatment effects of testosterone on prostate cancer (e.g. androgen deprivation therapy);
- Cell culture and animal studies presenting cell line data only;
- Observational studies where the exposure is measured within 2 years of the outcome to reduce the risk of reverse causality.

Peer-reviewed published articles, including supplements and meeting abstracts, are eligible sources of information about the studies. Systematic and non-systematic reviews, books, commentaries, and letters will be used to identify studies not identified within the database searches. There will be no restriction on language or publication date of articles.

### Search strategy

The following databases will be used to identify relevant published articles without year or language restrictions:

- PubMed (from inception to present)
- Ovid MEDLINE (from 1946 to present)
- Ovid EMBASE (from 1980 to present)
- BIOSIS Citation Index (1969 to present)

Two sets of searches will be performed: (1) studies that link the exposure of interest (vitamin D) to the intermediate phenotype (testosterone), and (2) studies that link the same intermediate phenotype to prostate cancer onset or progression. Search strategies will include standard controlled vocabulary (MeSH and Emtree), text words, and keywords. An example of the search strategy used in MEDLINE is shown in Additional file 2. The search strategy will be amended to accommodate the individual requirements of each database. An information specialist with experience of conducting systematic reviews will be consulted regarding the search strategy.

### Searching other resources

Relevant systematic reviews will be used to identify eligible studies not retrieved from the electronic searches. The reference lists of all included articles will be hand searched for additional studies. Subject experts will be contacted about any unpublished or published studies that were not yielded from the original search.

### Data management

References yielded from the literature searches will be imported first into Endnote for initial inspection. Duplicate references will be identified by importing each reference into Stata and analysing the title, author names, year of publication, and journal name for overlap. We will use EPPI-Reviewer [24] and other tools to assist with screening the references

### Selection of studies

Titles and abstracts of all articles yielded from the literature searches will be screened against the eligibility criteria. This process will be performed independently by two reviewers, who will base their decision for inclusion or exclusion on the criteria listed above. Where an abstract is not available, the full-text article will be reviewed. Any discrepancies found in the initial screening will be resolved through discussion with a third reviewer. The full text of potential articles for inclusion will also be screened independently with any discrepancies resolved through discussion. The reference list of all included articles will be searched for additional studies.

### Data extraction

The following data items will be extracted independently by two reviewers from the included studies using a predefined extraction tool:

- Publication information—article title, author details, publication type, year of study, location;
- Study population—sample size, demographics (age, ethnicity), cancer stage, comorbidities associated with testosterone levels (i.e. hypogonadism);
- Exposure or intervention—study or model design, intervention description (including type, dose and duration), length of follow-up;
- Outcomes—measures of serum or plasma level of total and/or free testosterone, and measures of prostate cancer progression (tumour stage, Gleason score, local and distant metastases, biochemical recurrence based on prostate-specific antigen [PSA] levels);
- Statistical measures—effect estimates (mean, standard deviation, *p* value, odds ratio, 95% confidence intervals); any model adjustments.

The extraction tool will be piloted by the two reviewers on a small sample of articles before the full extraction process is undertaken. Any disagreements found in all the data extractions will be resolved through discussion with a third reviewer.

### Quality assessment

Assessments of risk of bias in individual studies will be performed. Appropriate tools are available for different study designs involving humans [25–27]. To assess risk of bias in animal studies, the Systematic Review Centre for Laboratory animal Experimentation (SYRCLE) tool [28] will be used. Assessments will be performed independently by two reviewers with discrepancies resolved through discussion with a third reviewer. A summary table presenting risk of bias assessments for each study will be included in the review.

### Data synthesis and analysis

Data will be synthesised in accordance with the separate pathways (i.e. vitamin D-testosterone and testosterone-prostate cancer). Human and animal trials will be analysed separately. It is anticipated that there will be a large degree of heterogeneity between the studies, such as differences in study design (e.g. RCT, case-control), exposures (vitamin D, sunlight), measures, and samples. Meta-analyses will be performed only with studies that are sufficiently similar, using both random-effects and fixed-effect methods. Primary results will be from fixed-effects meta-analyses unless we observe strong evidence of heterogeneity in effect sizes and study sizes. The extent of statistical heterogeneity among the true effects across studies will be assessed using the between-study variance. If the studies cannot be judged to be answering comparable research questions, for example, if they differ substantially in methods, exposures, or outcomes, a narrative synthesis will be performed. Where possible, sub-group analysis will be performed to examine the sources of heterogeneity on study outcomes. Fixed-effect results will be presented graphically using forest plots. Alternatively, albatross plots [29] will be produced where effect sizes are not comparable.

### Level of certainty of evidence

The level of certainty of evidence provided from all studies will be assessed using the Grading of Recommendations Assessment, Development and Evaluation system (GRADE) [30]. GRADE classifies the confidence in an estimate into four levels: (1) high, (2) moderate, (3) low, and (4) very low. Estimates of effect can be either up or downgraded based on several of the following criteria:

- Study limitations (risk of bias);
- Inconsistency of results;
- Indirectness of evidence;
- Imprecision;
- Reporting bias.

A study rated as low certainty could be upgraded if the studies have reported a large magnitude of effect or a dose-response relationship, for example. Human and animal studies will be rated separately.

## Discussion

The aim of this review is to determine whether testosterone is a causally relevant intermediate mechanism underpinning the relationship between vitamin D and prostate cancer progression.

To our knowledge, this is the first review examining this mechanistic pathway in accordance with systematically synthesising the evidence of the relationship between vitamin D and testosterone. Other reviews have investigated the relationship between testosterone and prostate cancer outcomes, including increased risk in prostate cancer in hypogonadal men. However, many of these reviews tended to focus on the therapeutic use of testosterone to assess treatment efficacy for cancer [31, 32]. Here, we will review evidence from studies involving humans and experimental studies with rodents according to the WCRF International/University of Bristol methodology [15]. The synthesis of this evidence will suggest whether increase in vitamin D is a potential preventive measure of prostate cancer progression.

The search strategy and study inclusion criteria are relatively broad to capture the wealth of evidence which may exist. For human studies, no restrictions will be placed on population characteristics (i.e. co-morbidities, age), except where applicable (i.e. testosterone-prostate cancer relationship). Different modes of administration of the exposure variables (vitamin D, testosterone) and multiple outcomes of prostate cancer will be considered for inclusion in this review.

The review will exclude in vitro studies. These studies are beneficial to the understanding of the mechanistic pathway in terms of cellular and molecular activity. However, it is difficult for in vitro studies to mimic the complicated process that would occur within a natural biological context. They would also increase the heterogeneity of the synthesised results due to the number of different models that exist in these studies [33]. Therefore, human and in vivo animal studies will be considered for inclusion as their data will have more clinical relevance.

This review seeks to establish whether vitamin D is a potential therapeutic intervention for the prevention and/or progression of prostate cancer via its effects on testosterone levels. Based on its findings, the review may help inform future clinical intervention trials and other types of research, such as public health messages.

## Additional files

Additional file 1: PRISMA-P 2015 Checklist. (DOCX 30 kb)

Additional file 2: MEDLINE search strategy. (DOCX 15 kb)

## Abbreviations

GRADE: Grading of Recommendations Assessment, Development and Evaluation system
MELODI: Mining Enriched Literature Objects to Derive Intermediates
PRISMA-P: Preferred Reporting Items for Systematic review and Meta-Analysis Protocols
RNA: Ribonucleic acid
SYRCLE: Systematic Review Centre for Laboratory animal Experimentation
TeMMPo: Text Mining for Mechanism Prioritisation
