# Supplementary material for: Does testosterone mediate the relationship between vitamin D and prostate cancer? A systematic review and meta-analysis protocol
Source: Syst Rev. 2019 Feb 12;8:52. doi: 10.1186/s13643-018-0908-1 (PMC6371501; doi:10.1186/s13643-018-0908-1)
Supplement: Supplementary file 2 — MEDLINE search strategy. (DOCX 15 kb) [file 13643_2018_908_MOESM2_ESM.docx]

**MEDLINE search strategy**

1. exp Vitamin D/

2. vitamin D?.ti,ab,kf.

3. Vitamin D?.hw.

4. exp Ergocalciferols/

5. ergocalciferols.ti,ab,kf.

6. exp Cholecalciferol/

7. cholecalciferol.ti,ab,kf.

8. exp Calcitriol/

9. calcitriol.ti,ab,kf.

10. Receptors, Calcitriol/

11. vitamin D receptor.ti,ab,kf.

12. (hydroxycholecalciferol? or hydroxy-cholecalciferol? or dihydroxycholecalciferol? or dihydroxy-cholecalciferol? or hydroxyvitamin? D? or hydroxyl-vitamin? D?).ti,ab,kf.

13. 1 or 2 or 3 or 4 or 5 or 6 or 7 or 8 or 9 or 10 or 11 or 12

14. exp Testosterone/

15. testosterone.ti,ab,kf.

16. *Gonadal Hormones/

17. *Gonadal Steroid Hormones/

18. exp Testicular Hormones/

19. exp Testosterone Congeners/

20. 14 or 15 or 16 or 17 or 18 or 19

21. exp Prostatic Neoplasms/

22. (prostat$ adj3 (neoplas$ or cancer$ or carcinoma$ or adenocarcinoma$ or tumo?r$)).ti,ab.

23. (prostat$ and (neoplas$ or cancer$ or carcinoma$ or adenocarcinoma$ or tumo?r$)).kf.

24. 22 or 23

25. exp Neoplasm Metastasis/

26. exp Neoplasm Invasiveness/

27. ((metasta* or (neoplas* or cancer* or carcinoma* or adenocarcinoma* or tumo?r*)) and (invas* or invad* or aggressive* or progressi*)).ti,kf.

28. tumo?r progression.ti,kf.

29. 25 or 26 or 27 or 28

30. exp Prostatic Intraepithelial Neoplasia/

31. 21 or 24 or 30

32. 29 and 31

33. 13 and 20

59. 20 and 32
